# Aluminum exposure impairs nuclear envelope breakdown for mouse zygote formation

**DOI:** 10.1016/j.isci.2026.115807

**Published:** 2026-04-21

**Authors:** Xiao-Ting Yu, Xing-He Ke, Zi-Jian Wu, Zhen-Hui Fu, Rui-Jie Ma, Xuan Wu, Meng-Meng Tang, Shao-Chen Sun, Lin-Lin Hu

**Affiliations:** 1 Key Laboratory of Research on Clinical Molecular Diagnosis for High Incidence Diseases in Western Guangxi of Guangxi Higher Education Institutions, Reproductive Medicine of Guangxi Medical and Health Key Discipline Construction Project, Affiliated Hospital of Youjiang Medical University for Nationalities, Baise 533000, China; 2 College of Animal Science and Technology, Nanjing Agricultural University, Nanjing 210095, China

**Keywords:** environmental toxicology, cell biology, developmental biology

## Abstract

Aluminum exposure ubiquitously presents in the environment and its toxicity elicits oxidative stress and metabolic disorders. In the present study, we reported that aluminum exposure disturbed zygote formation with mouse model. We found that aluminum-exposed mouse zygotes exhibited nuclear envelope breakdown defects, indicative of failed zygote development. Further analyses revealed that microtubules decreased with aberrant tubulin acetylation, and actin dynamics was disturbed, indicating the cytoskeleton impairment. This further leads to DNA damage and disturbed cell cycle in zygotes. Moreover, mitochondrial dysfunction was observed, which triggers oxidative damage and apoptosis. These defects were closely linked to the aberrant distribution and function of the organelles for protein modification and intracellular transport in zygotes. Collectively, our results indicate that aluminum exposure compromises cytoskeletal integrity and cell cycle progression, which may be due to mitochondria-based oxidative damage and apoptosis for organelle function, and ultimately disrupts nuclear envelope breakdown for zygote formation in mice.

## Highlights

• Aluminum causes nuclear envelope breakdown defects during zygote formation
• Aluminum exposure impairs cytoskeleton integrity and causes cell-cycle arrest
• Aluminum induces mitochondrial dysfunction-based oxidative damage and apoptosis
• Aluminum disrupts the distribution and function of organelles in zygotes

## Graphical abstract

**Figure:**
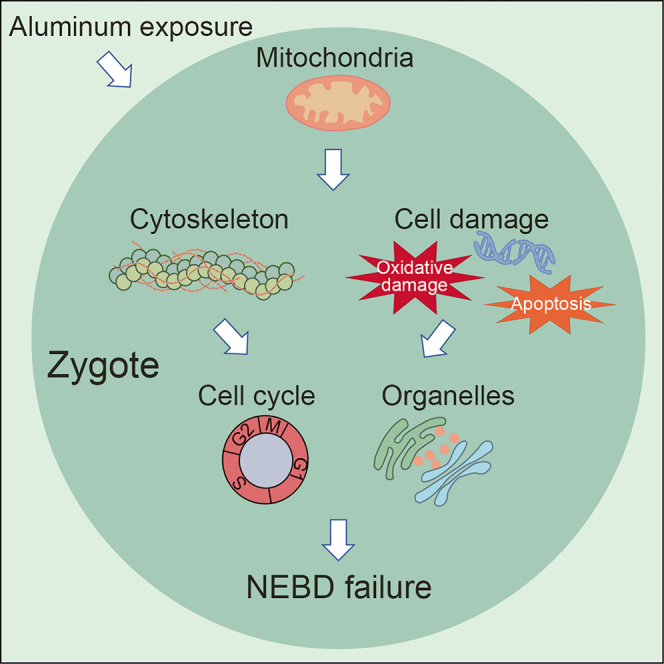

## Introduction

Aluminum is a silver-white metal with a lustrous surface. As the most abundant metallic element in the Earth’s crust, it primarily exists in compound forms.^1^ Humans are exposed to aluminum through diverse pathways, with dietary intake^2^ and dermal contact^3^ being the primary routes. Additional exposure routes include inhalation of aluminum-containing dust/fumes via the respiratory tract, consumption of aluminum-based medications, and occupational exposure in specific industries.^4^ Aluminum has extensive applications, ranging from packaging materials and construction components to household items such as cookware and furniture.^5^ Prolonged aluminum exposure induces toxicity across multiple organ systems. Animal studies show that aluminum impairs cognitive function in mice by causing memory deficits and neuroinflammation.^6^ In humans, aluminum accumulation is linked to neurotoxicity, contributing to brain damage and an increased risk of Alzheimer’s disease.^7^ Chronic exposure disrupts vascular function by reducing vasoreactivity and nitric oxide bioavailability, leading to vascular dysfunction and hypertension.^8^ In the lungs, aluminum exposure triggers inflammation, fibrosis, impaired pulmonary function, and oxidative stress injury, with long-term occupational exposure elevating the risk of respiratory diseases.^9^ Skeletal disorders may arise from aluminum deposition in bones, particularly in patients with chronic kidney disease.^10^ Aluminum also interferes with iron transport/utilization, causing iron deficiency during erythropoiesis and microcytic anemia.^11^ Additionally, it impairs intestinal function by inducing oxidative stress and inflammatory responses, disrupting tight junctions in intestinal epithelial cells.^12^

Aluminum, a ubiquitous metal, has been widely documented for its reproductive toxicity. In males, it disrupts spermatogenesis, causing disordered cell arrangement, expanded intercellular spaces, and reduced sperm count in seminiferous tubules.^13^ Aluminum exposure in male rats impairs intracellular redox homeostasis by elevating reactive oxygen species (ROS) production and decreasing antioxidant enzyme activity, thereby activating apoptotic pathways, and increasing testicular cell apoptosis.^14^ Additionally, it significantly reduces testis and epididymis size, decreases testosterone and luteinizing hormone (LH) levels, and induces necrosis in seminiferous tubule epithelium, Leydig cells, and the basal epithelium of the epididymis and prostate.^15^ In females, aluminum exposure compromises ovarian architecture, reduces enzymatic activity, disrupts iron/zinc/copper metabolism, and downregulates follicle-stimulating hormone receptor (FSHR) and LH receptor (LHR) expression. This results in insufficient ovarian energy supply, suppressed ovulation and luteal development, and infertility.^16^ Morphological alterations include impaired follicular development, increased follicular atresia, reduced corpus luteum count, and ovarian stromal cell proliferation.^17^ Aluminum also impairs oocyte quality by disrupting cytoskeletal integrity, disturbing organelle function, dysregulating calcium homeostasis, inducing oxidative stress, and causing DNA damage-mediated apoptosis.^18^ However, currently, there are only a few studies on the mechanisms underlying the embryotoxic effects of aluminum exposure.

Embryonic development is a highly orchestrated process spanning from the zygote stage to embryonic maturation, requiring precise cell proliferation, differentiation, and coordinated intercellular interactions.^19^ At fertilization, a mature sperm fuses with an MII-arrested oocyte, forming male and female pronuclei that migrate centrally via microfilament-microtubule interactions, culminating in nuclear envelope breakdown (NEBD).^20^ The zygote then undergoes cleavage to form a blastocyst, composed of an outer trophoblast, an inner cell mass (ICM), and a hypoblast layer. The ICM differentiates into epiblast and hypoblast.^21^ Nuclear actin supports embryonic development by regulating chromatin organization, gene expression, and DNA damage responses (DDRs). Its dynamic remodeling is critical for early embryo development and cell fate determination.^22^ DNA damage activates CHK1 protein kinase, initiating cell cycle checkpoint signaling to arrest progression.^23^ CHK1 regulates the cell cycle by degrading CDC25A to inhibit CDK1 activity. Depletion or inhibition of CHK1 leads to DNA damage, shortened G2 phase, and blocked genome activation, causing embryonic arrest and infertility in mice.^24^ Mitochondrial dysfunction promotes ROS production, disrupting the oxidative-antioxidant balance and inducing oxidative stress.^25^ Elevated ROS oxidizes cellular nucleic acids, proteins, and lipids, leading to growth arrest and cell death.^26^ Oxidative stress triggers endoplasmic reticulum (ER) stress to activate apoptotic factors such as Bcl-2, and excessive inhibition of Bcl-2’s anti-apoptotic function enhances cell apoptosis, thereby impairing embryonic development.^27^ Lysosomal dysfunction leads to intracellular accumulation of waste and damage signals, inducing apoptosis and arresting embryonic development.^28^

Using *in vitro* fertilized (IVF) mouse zygotes as models, this study exposed early-stage zygotes to aluminum chloride (AlCl_3_). Results show that aluminum exposure compromises cytoskeletal integrity, disrupts cell cycle progression, induces oxidative damage and apoptosis, impairs cellular functions, and ultimately inhibits NEBD in zygotes, thereby arresting embryonic development.

## Results

### Aluminum exposure causes failure of nuclear envelope breakdown in mouse zygotes

**Figure 1:**
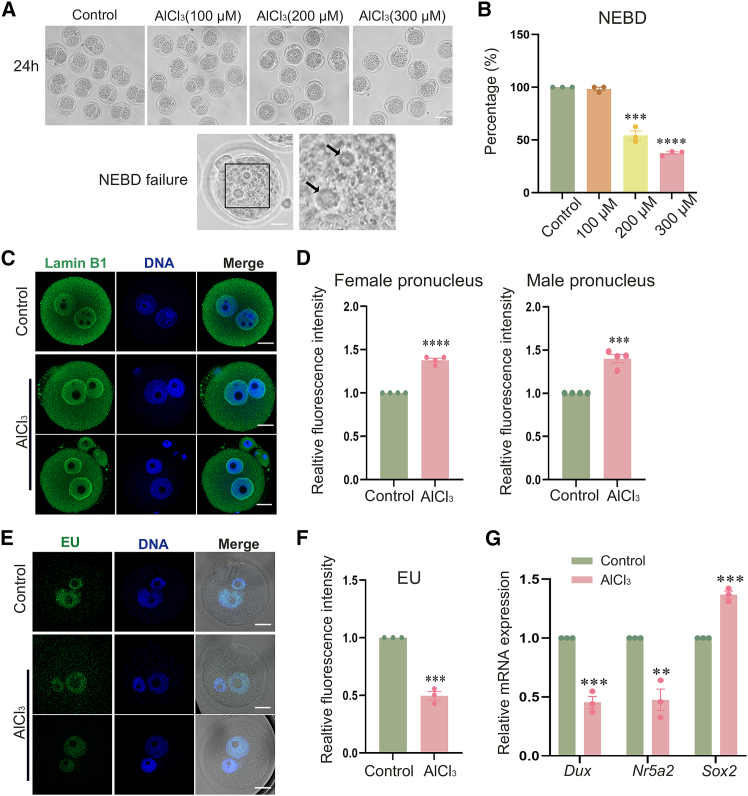
Aluminum exposure leads to nuclear envelope breakdown failure in mouse zygotes (A) Typical images of embryonic development in mouse zygotes treated with different concentrations of AlCl_3_ at 24 h postfertilization. Scale bars, 80 μm; scale bars, 20 μm. (B) The effect of different AlCl_3_ concentrations on nuclear envelope breakdown rate in mouse zygotes. Control, *n* = 75; AlCl_3_ (100 μM), *n* = 66; AlCl_3_ (200 μM), *n* = 105, ∗∗∗*p* < 0.0005; AlCl_3_ (300 μM), *n* = 69, ∗∗∗∗*p* < 0.0001 (based on the results of the concentration gradient assay, 300 μM AlCl_3_ was selected for subsequent experiments. Therefore, only AlCl_3_ is indicated in the following figures/data without specifying the concentration each time). (C) Typical images of Lamin B1 protein fluorescence signal in the control group and AlCl_3_-treated group by immunofluorescence staining. Green, Lamin B1; blue, DNA. Scale bars, 20 μm. (D) Relative fluorescence intensity of Lamin B1 protein in female pronucleus and male pronucleus. Female pronucleus: control, *n* = 35; AlCl_3_, *n* = 44, ∗∗∗∗*p* < 0.0001; male pronucleus: control, *n* = 35; AlCl_3_, *n* = 44, ∗∗∗*p* < 0.0005. (E) Typical images of EU signal in the control group and AlCl_3_-treated mouse zygotes by EU staining. Green, EU; blue, DNA. Scale bars, 20 μm. (F) Relative fluorescence intensity of EU in the control group and AlCl_3_-treated group. Control, *n* = 36; AlCl_3_, *n* = 42, ∗∗∗*p* < 0.0005. (G) The mRNA expression levels of transcription factors in zygotes from control and treatment groups were determined by RT-qPCR. ∗∗∗*p* < 0.0005, ∗∗*p* < 0.005, ∗∗∗*p* < 0.0005. Data are presented as mean ± SEM. All experiments were performed in three independent biological replicates, with three technical replicates per biological replicate; mouse zygotes were used as research subjects. *n* represents the number of zygotes analyzed. Statistical analysis was performed using paired *t* tests. Asterisks indicate statistical significance: ∗*p* < 0.05, ∗∗*p* < 0.01, ∗∗∗*p* < 0.001, ∗∗∗∗*p* < 0.0001.

**Figure 2:**
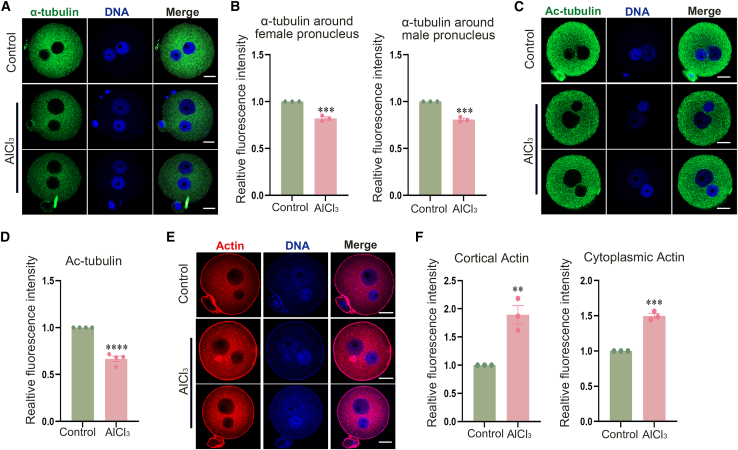
Aluminum exposure disrupts microtubule and actin filament structures in mouse zygotes (A) Typical images of the distribution of α-tubulin in control and AlCl_3_-treated mouse zygotes by immunofluorescence staining. Green, α-tubulin; blue, DNA. Scale bars, 20 μm. (B) Relative fluorescence intensity of α-tubulin around female and male pronuclei. Female pronucleus: Control, *n* = 42; AlCl_3_, *n* = 52, ∗∗∗*p* < 0.001; Male pronucleus: control, *n* = 42; AlCl_3_, *n* = 52, ∗∗∗*p* < 0.0005. (C) Typical images of Ac-tubulin staining in control and treatment groups by immunofluorescence staining. Green, Ac-tubulin; blue, DNA. Scale bars, 20 μm. (D) Relative fluorescence intensity of Ac-tubulin. Control, *n* = 56; AlCl_3_, *n* = 50, ∗∗∗∗*p* < 0.0001. (E) Typical distribution of actin in control and AlCl_3_-treated mouse zygotes by fluorescence staining. Red, actin; blue, DNA. Scale bars, 20 μm. (F) Relative fluorescence intensity of cortical actin and cytoplasmic actin. Cortical actin: control, *n* = 42; AlCl_3_, *n* = 43, ∗∗*p* < 0.01; cytoplasmic actin: control, *n* = 41; AlCl_3_, *n* = 44, ∗∗∗*p* < 0.0005. Data are presented as mean ± SEM. All experiments were performed in three independent biological replicates, with three technical replicates per biological replicate; mouse zygotes were used as research subjects. *n* represents the number of zygotes analyzed. Statistical analysis was performed using paired *t* tests. Asterisks indicate statistical significance: ∗*p* < 0.05, ∗∗*p* < 0.01, ∗∗∗*p* < 0.001, ∗∗∗∗*p* < 0.0001.

To investigate the potential effects of aluminum on early mouse embryonic development, this study first evaluated embryos treated with 100 μM, 200 μM, and 300 μM AlCl_3_ and quantified the NEBD rate at 24 h postfertilization. As depicted in Figures 1A and 1B, most embryos cultured in 200 μM and 300 μM AlCl_3_ for 24 h failed to undergo NEBD, whereas most embryos exposed to 100 μM AlCl_3_ successfully reached the two-cell stage. In developmentally arrested zygotes, the persistent presence of both male and female pronuclei indicated that aluminum exposure disrupted normal NEBD. Additionally, the rate of NEBD in the AlCl3-treated group was significantly lower than that observed in the control group (control, 100 ± 0.00%, *n* = 75 vs. AlCl_3_ (100 μM), 98.33 ± 1.67%, *n* = 66 vs. AlCl_3_ (200 μM), 54.33 ± 4.29%, *n* = 105, *p* < 0.0005, AlCl_3_ (300 μM), 37.53 ± 1.28%, *n* = 69, *p* < 0.0001). Therefore, in this study, AlCl_3_ with a concentration of 300 μM was used for the subsequent experiments. To further investigate the impact of aluminum on the nuclear envelope of zygotes, we performed Lamin B1 immunofluorescence staining on embryos fixed at 12 h postfertilization. As depicted in Figure 2C, the fluorescence intensity of Lamin B1 in the female and male pronucleus was significantly elevated in the AlCl_3_-treated group compared to controls. As shown in Figures 2D and 2E, the relative fluorescence intensities of Lamin B1 in the female and male pronuclei of the AlCl_3_ treatment group were significantly higher than those in the control group (female pronucleus: control, 1.00 ± 0.00, *n* = 35 vs. AlCl_3_, 1.38 ± 0.02, *n* = 44, *p* < 0.0001; male pronucleus: control, 1.00 ± 0.00, *n* = 35 vs. AlCl_3_, 1.4 ± 0.05, *n* = 44, *p* < 0.0005). To further investigate the effects of aluminum on transcription in zygotes, we performed 5-ethynyl uridine (EU) staining to quantify nascent RNA synthesis. As shown in Figures 1E and 1F, EU fluorescence intensity was significantly reduced in AlCl_3_-treated embryos compared to controls, as confirmed by quantitative image analysis (control, 1.00 ± 0.00, *n* = 36; AlCl_3_, 0.50 ± 0.04, *n* = 42; *p* < 0.0005). Additionally, we evaluated the mRNA expression levels of several transcription factors using RT-qPCR, including *Dux*, *Nr5a2*, and *Sox2*. As shown in Figure 1G, the expression levels of *Dux* (0.45 ± 0.05 vs. 1, *p* < 0.0005), *Nr5a2* (0.48 ± 0.09 vs. 1, *p* < 0.005) were significantly diminished compared with the control group, and the expression levels of *Sox2* (1.37 ± 0.03 vs. 1, *p* < 0.0005) were elevated compared with the control group. In conclusion, this study demonstrates that high concentrations of aluminum cause failure of NEBD in zygotes by disrupting nuclear membrane structural integrity and inhibiting transcriptional activity, thereby arresting early embryonic development.

### Aluminum exposure disrupts microtubule and actin filament dynamics in mouse zygotes

To investigate the effect of aluminum on microtubule organization, we performed α-tubulin immunofluorescence staining in zygotes. As visualized in Figure 2A, microtubules in control embryos were predominantly localized around the female and male pronuclei and throughout the cytoplasm. Then we conducted a statistical analysis of fluorescence intensity. As shown in Figure 2B, the results showed that the relative fluorescence of α-tubulin around the prokaryotes of females and males was significantly reduced (female pronucleus: control, 1.00 ± 0.00%, *n* = 42 vs. AlCl_3_, 0.82 ± 0.02, *n* = 52, *p* < 0.001; male pronucleus: control, 1.00 ± 0.00, *n* = 42 vs. AlCl_3_, 0.81 ± 0.01, *n* = 52, *p* < 0.0005). After that, we measured the fluorescence intensity of α-tubulin acetylation, which is associated with microtubule stability. As depicted in Figures 2C and 2D, the relative fluorescence intensity of AC-tubulin was significantly reduced in the treatment group than in the control group (control, 1.00 ± 0.00, *n* = 56; AlCl_3_, 0.67 ± 0.03, *n* = 50; *p* < 0.0001). Actin plays an essential role in the migration and precise positioning of pronuclei. Phalloidin-TRITC staining was performed to visualize actin filaments in zygotes. As illustrated in Figures 2E and 2F, during the zygote stage, actin is localized in both the cortex and cytoplasm. In the AlCl_3_-treated group, the relative fluorescence intensity of Actin in both the cortex and cytoplasm was significantly higher than that in the control group (cortical actin: control, 1.00 ± 0.00, *n* = 42; AlCl_3_, 1.89 ± 0.16, *n* = 43; *p* < 0.01; cytoplasmic actin: control, 1.00 ± 0.00, *n* = 41; AlCl_3_, 1.50 ± 0.04, *n* = 44; *p* < 0.0005). These findings indicate that aluminum exposure reduces microtubule density and disrupts actin assembly around pronuclei in mouse zygotes.

### Aluminum exposure disturbs cell cycle progression in mouse zygotes

**Figure 3:**
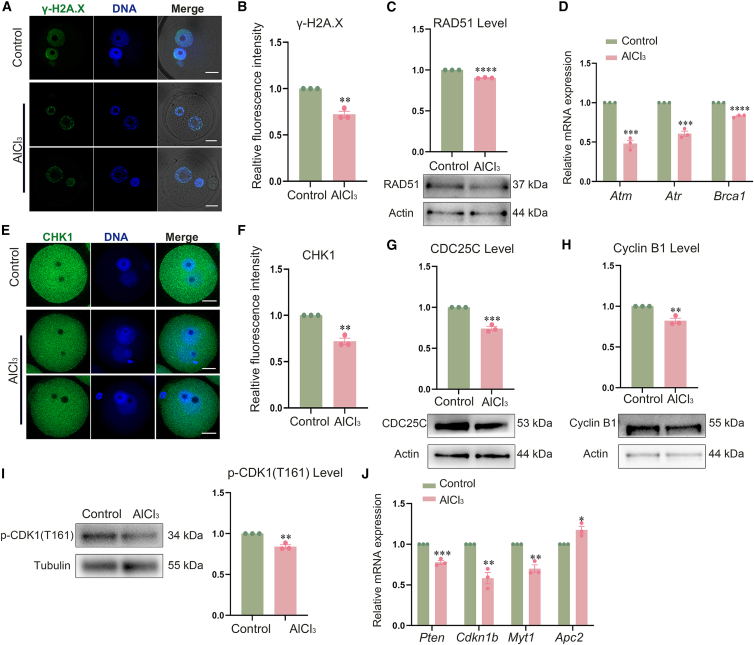
Aluminum exposure induces G2/M phase arrest in mouse zygotes (A) Representative images of γ-H2A.X in zygotes from the control group and the AlCl_3_-treated group by immunofluorescence staining. Green, γ-H2A.X; blue, DNA. Scale bars, 20 μm. (B) Relative fluorescence intensity of γ-H2A.X. Control, *n* = 40; AlCl_3_, *n* = 41; ∗∗*p* < 0.005. (C) Expression level and statistical analysis of RAD51 protein in zygotes of the control group and AlCl_3_-treated group by western blot. ∗∗∗∗*p* < 0.0001. (D) The mRNA expression levels of DNA damage-related genes were significantly downregulated in the treatment group compared with the control by RT-qPCR. ∗∗∗*p* < 0.0005, ∗∗∗*p* < 0.0005, ∗∗∗∗*p* < 0.0001. (E) Representative fluorescence images of CHK1 signals in zygotes of the control group and AlCl_3_-treated group by immunofluorescence staining. Green, CHK1; blue, DNA. Scale bars, 20 μm. (F) Relative fluorescence intensity of CHK1. Control, *n* = 34; AlCl_3_, *n* = 33; ∗∗*p* < 0.005. (G) Expression level and statistical analysis of CDC25C protein in zygotes of the control group and AlCl_3_-treated group by western blot. ∗∗∗*p* < 0.001. (H) Expression level and statistical analysis of Cyclin B1 protein in zygotes of the control group and AlCl_3_-treated group by western blot. ∗∗*p* < 0.01. (I) Expression level and statistical analysis of *p*-CDK1(T161) protein in zygotes of the control group and AlCl_3_-treated group by western blot. ∗∗*p* < 0.005. (J) The mRNA expression of cell cycle regulatory genes by RT-qPCR. ∗∗∗*p* < 0.001, ∗∗*p* < 0.005, ∗∗*p* < 0.005, ∗*p* < 0.05. Data are presented as mean ± SEM. All experiments were performed in three independent biological replicates, with three technical replicates per biological replicate; mouse zygotes were used as research subjects. *n* represents the number of zygotes analyzed. Statistical analysis was performed using paired *t* tests. Asterisks indicate statistical significance: ∗*p* < 0.05, ∗∗*p* < 0.01, ∗∗∗*p* < 0.001, ∗∗∗∗*p* < 0.0001.

In mouse zygotes, the normal progression of the G2/M phase, which is critical for preparing cells for mitosis, is essential for the timely occurrence of NEBD, a key event that initiates mitosis. Conversely, DNA damage induces G2 phase arrest in these embryos, thereby blocking their transition to the M phase. To investigate this further, we first examined the fluorescence intensity of the DNA damage marker γ-H2A.X. As shown in Figures 3A and 3B, the relative fluorescence intensity of γ-H2A.X was found to decrease (control, 1.00 ± 0.00, *n* = 40; AlCl_3_, 0.72 ± 0.03, *n* = 41; *p* < 0.005). Next, we detected the expression level of RAD51, a protein associated with DNA damage repair. As shown in 3C, the results showed that the expression level of RAD51 was decreased in the AlCl_3_-treated group (0.90 ± 0.003 vs. 1, *p* < 0.0001). Then we examined the mRNA expression levels of DNA damage-related genes. As shown in Figure 3D, the mRNA expression levels of *Atm* (0.48 ± 0.04 vs. 1, *p* < 0.0005), *Atr* (0.60 ± 0.03 vs. 1, *p* < 0.0005), and *Brca1* (0.83 ± 0.01 vs. 1, *p* < 0.0001) were found to be decreased. As a key DNA damage checkpoint protein, CHK1 is intimately involved in cell cycle regulation. To assess DDR signaling, CHK1 immunofluorescence staining was performed. As depicted in Figures 3E and 3F, the relative fluorescence intensity of CHK1 was notably reduced in the AlCl_3_-treated group (control, 1.00 ± 0.00, *n* = 34; AlCl_3_, 0.72 ± 0.03, *n* = 33; *p* < 0.005). Subsequently, we conducted western blotting to examine the protein levels of CDC25C, cyclin B1, and *p*-CDK1(T161). The results are depicted in Figures 3G–3I. In the AlCl_3_ treatment group, the protein levels of CDC25C (0.74 ± 0.02 vs. 1, *p* < 0.001), cyclin B1 (0.82 ± 0.03 vs. 1, *p* < 0.01), and *p*-CDK1(T161) (0.84 ± 0.02 vs. 1, *p* < 0.005) were found to be significantly reduced. Additionally, we evaluated the mRNA expression levels of cell cycle regulators *Pten*, *Cdkn1b*, *Myt1*, and *Apc2* using RT-qPCR. As illustrated in Figure 3J, the mRNA expression levels of *Pten* (0.78 ± 0.02 vs. 1, *p* < 0.001), *Cdkn1b* (0.58 ± 0.07 vs. 1, *p* < 0.005), and *Myt1* (0.70 ± 0.05 vs. 1, *p* < 0.005) was found to be decreased, whereas that of *Apc2* (1.18 ± 0.05 vs. 1, *p* < 0.05) were found to be increased. This result suggests that aluminum chloride exposure impairs DNA damage sensing and repair capacity and disrupts CHK1-mediated G2/M checkpoint regulation, thereby inducing G2-phase arrest and blocking mitotic entry in mouse zygotes.

### Aluminum exposure induces mitochondrial dysfunction, oxidative damage accumulation and apoptosis in mouse zygotes

**Figure 4:**
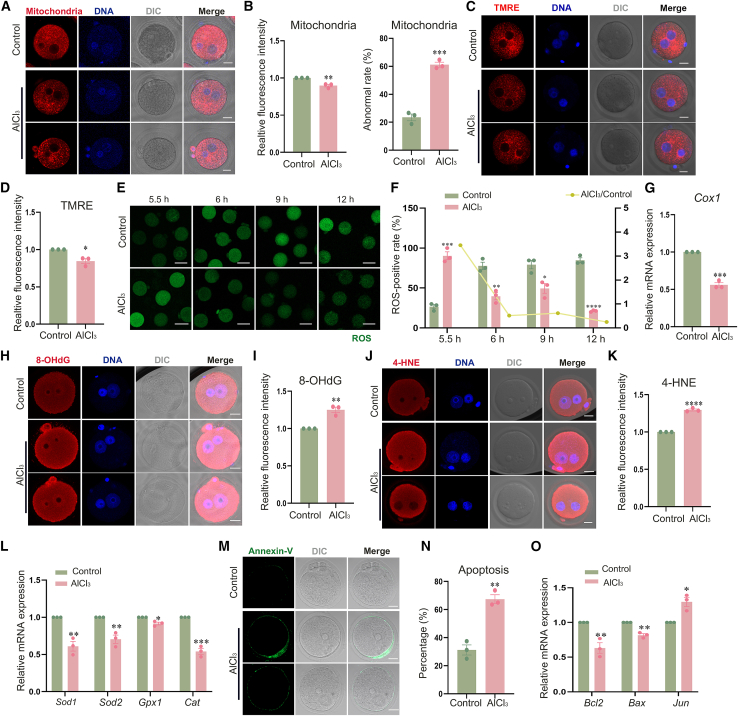
Aluminum exposure triggers mitochondrial dysfunction, oxidative damage accumulation and apoptosis in mouse zygotes (A) Representative images of mitochondrial distribution in zygotes of the control and treatment groups by immunofluorescence staining. Red, mitochondria; blue, DNA. Scale bars, 20 μm. (B) Relative fluorescence intensity and abnormal rate of mitochondria. The relative fluorescence intensity: control, *n* = 47; AlCl_3_, *n* = 50; ∗∗*p* < 0.005; abnormal rate: control, *n* = 47; AlCl_3_, *n* = 50; ∗∗∗*p* < 0.0005. (C) TMRE levels in zygotes of the control and treatment groups by immunofluorescence staining. Red, TMRE; blue, DNA. Scale bars, 20 μm. (D) Relative fluorescence intensity of TMRE. Control, *n* = 45; AlCl_3_, *n* = 46; ∗*p* < 0.05. (E) ROS-positive rate in zygotes of the control and treatment groups at 5.5 h, 6 h, 9 h, and 12 h post-fertilization by immunofluorescence staining. Green, ROS. Scale bars, 80 μm. (5.5 h corresponds to 30 min after AlCl_3_ treatment, 6 h to 1 h, 9 h–4 h, and 12 h–7 h after AlCl_3_ treatment). (F) ROS-positive rate in zygotes at different time points post-fertilization. 5.5 h: control, *n* = 39; AlCl_3_, *n* = 38; ∗∗∗*p* < 0.0005; 6 h: control, *n* = 45; AlCl_3_, *n* = 47; ∗∗*p* < 0.005; 9 h: control, *n* = 41; AlCl_3_, *n* = 42; ∗*p* < 0.05; 12 h: control, *n* = 38; AlCl_3_, *n* = 46; ∗∗∗∗*p* < 0.0001. (G) The mRNA expression level of *Cox1*. ∗∗∗*p* < 0.0005. (H) 8-OHdG levels in zygotes of the control and treatment groups by immunofluorescence staining. Red, 8-OHdG; blue, DNA. Scale bars, 20 μm. (I) Relative fluorescence intensity of 8-OHdG. Control, *n* = 41; AlCl_3_, *n* = 39; ∗∗*p* < 0.005. (J) The 4-HNE levels in zygotes of the control and treatment groups by immunofluorescence staining. Red, 4-HNE; blue, DNA. Scale bars, 20 μm. (K) Relative fluorescence intensity of 4-HNE. Control, *n* = 39; AlCl_3_, *n* = 41; ∗∗∗∗*p* < 0.0001. (L) The mRNA expression levels of oxidative stress-related genes. ∗∗*p* < 0.005, ∗∗*p* < 0.01, ∗*p* < 0.05, ∗∗∗*p* < 0.0005. (M) Representative images of early apoptosis in control and AlCl_3_-exposed groups by immunofluorescence staining. Green, annexin-V. Scale bars, 20 μm. (N) Apoptosis rate of zygotes. Control, *n* = 56; AlCl_3_, *n* = 50; ∗∗*p* < 0.005. (O) The mRNA expression levels of apoptosis-related genes. ∗∗*p* < 0.01, ∗∗*p* < 0.005, ∗*p* < 0.05. Data are presented as mean ± SEM. All experiments were performed in three independent biological replicates, with three technical replicates per biological replicate; mouse zygotes were used as research subjects. *n* represents the number of zygotes analyzed. Statistical analysis was performed using paired *t* tests. Asterisks indicate statistical significance: ∗*p* < 0.05, ∗∗*p* < 0.01, ∗∗∗*p* < 0.001, ∗∗∗∗*p* < 0.0001.

Mitochondrial dysfunction induces oxidative stress, generating excessive ROS that damage DNA, proteins, and lipids in the embryo, thereby disrupting the normal physiological functions of the cells and early developmental block at the 1-cell stage. Mito-Tracker staining was used to examine the effect of aluminum exposure on mitochondrial morphology and distribution in zygotes. As shown in Figures 4A and 4B, mitochondria in control embryos exhibited uniform cytoplasmic distribution and perinuclear aggregation. By contrast, AlCl_3_-treated embryos displayed aberrant mitochondrial localization, characterized by irregular dispersion of the organelles throughout the cytoplasm and a marked reduction in relative fluorescence intensity (control, 1.00 ± 0.00, *n* = 47; AlCl_3_, 0.90 ± 0.02, *n* = 50; *p* < 0.005). Consistently, the incidence of abnormal mitochondrial distribution was significantly higher in the AlCl_3_-treated group than in the control group (control, 23.47 ± 2.41%, *n* = 47 vs. AlCl_3_, 61.18 ± 1.80%, *n* = 50, *p* < 0.0005). Next, TMRE staining was employed to evaluate the mitochondrial membrane potential in mouse zygotes. As shown in Figures 4C and 4D, compared with the control group, the TMRE fluorescence intensity in the aluminum-treated group was significantly decreased (control, 1.00 ± 0.00, *n* = 45; AlCl_3_, 0.85 ± 0.03, *n* = 46; *p* < 0.05). After that, we evaluated the ROS positive rate at 5.5 h, 6 h, 9 h, and 12 h post-fertilization. As shown in Figures 4E and 4F, in AlCl_3_-treated embryos, the ROS-positive rate was increased at 5.5 h post-fertilization (control, 26.67 ± 2.65%, *n* = 39; AlCl_3_, 90.28 ± 5.01%, *n* = 38; *p* < 0.0005), while a decline in ROS-positive rate was observed at 6 h (control, 77.70 ± 4.60%, *n* = 45; AlCl_3_, 39.70 ± 4.51%, *n* = 47; *p* < 0.005), 9 h (control, 79.33 ± 5.29%, *n* = 41; AlCl_3_, 49.50 ± 6.07%, *n* = 42; *p* < 0.05), and 12 h (control, 84.66 ± 3.13%, *n* = 38; AlCl_3_, 21.22 ± 0.65%, *n* = 46; *p* < 0.0001) post-fertilization. We measured the mRNA expression levels of *Cox1* via RT-qPCR. As shown in Figure 4G, the mRNA expression levels of *Cox1* (0.56 ± 0.03 vs. 1, *p* < 0.0005) were decreased. After that, we measured the immunofluorescence intensity of the DNA oxidative damage product 8-hydroxy-2′-deoxyguanosine (8-OHdG) and the lipid oxidative damage product 4-hydroxynonenal (4-HNE) in both the control and AlCl_3_-treated embryos. As shown in Figures 4H and 4I, the relative immunofluorescence intensity of 8-OHdG was significantly elevated in the AlCl_3_-treated group compared with the control group (control, 1.00 ± 0.00, *n* = 41; AlCl_3_, 1.25 ± 0.04, *n* = 39; *p* < 0.005). As shown in Figures 4J and 4K, the relative immunofluorescence intensity of 4-HNE was significantly elevated in the AlCl_3_-treated group compared with the control group (control, 1.00 ± 0.00, *n* = 39; AlCl_3_, 1.30 ± 0.01, *n* = 41; *p* < 0.0001). We further quantified the mRNA expression levels of oxidative stress-associated proteins via RT-qPCR. As shown in Figure 4L, the mRNA expression levels of *Sod1* (0.61 ± 0.06 vs. 1, *p* < 0.005), *Sod2* (0.71 ± 0.06 vs. 1, *p* < 0.01), *Gpx1* (0.92 ± 0.02 vs. 1, *p* < 0.05), and *Cat* (0.54 ± 0.04 vs. 1, *p* < 0.0005) were all decreased. Oxidative damage induces embryonic apoptosis through ROS-mediated biomolecular damage, including lipid peroxidation, protein dysfunction, and DNA injury. This damage activates apoptotic signaling pathways, ultimately impairing normal embryonic development. To confirm that, we examined the apoptosis rate using Annexin-V staining. As shown in 4M and N, compared with the control group, the apoptosis rate in the AlCl_3_-treated group was significantly increased (control, 31.25 ± 3.61%, *n* = 56 vs. AlCl_3_, 67.44 ± 3.15%, *n* = 50, *p* < 0.005). Furthermore, we quantified the mRNA expression levels of apoptosis-related genes using RT-qPCR, as shown in Figure 4O, the mRNA expression levels of *Bcl2* (0.63 ± 0.08 vs. 1, *p* < 0.01) and *Bax* (0.82 ± 0.02 vs. 1, *p* < 0.005) were both downregulated and *Jun* (1.30 ± 0.07, *p* < 0.05) was upregulated compared to the control group. These findings indicate that aluminum exposure impairs mitochondrial dysfunction and subsequent oxidative damage and apoptosis in aluminum-exposed mouse zygotes.

### Aluminum exposure disrupts the organelle functions in mouse zygotes

**Figure 5:**
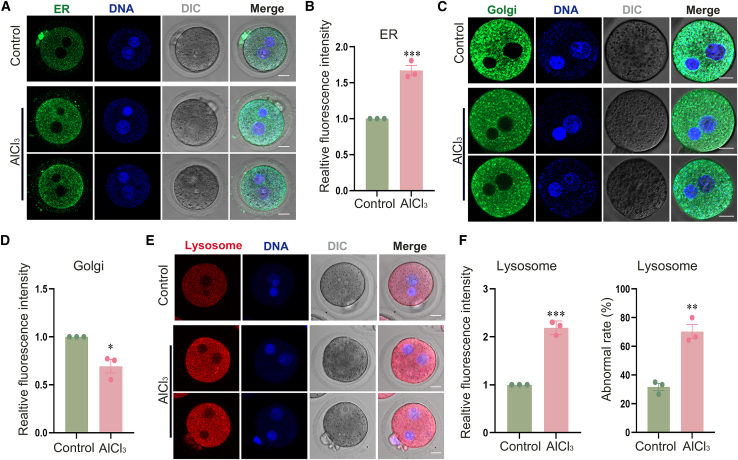
Aluminum exposure induces functional abnormalities in the endoplasmic reticulum, Golgi apparatus, and lysosomes in mouse zygotes (A) Representative images of endoplasmic reticulum (ER) distribution in zygotes of the control and treatment groups by immunofluorescence staining. Green, ER; blue, DNA. Scale bars, 20 μm. (B) Relative fluorescence intensity of ER in zygotes. Control, *n* = 52; AlCl_3_, *n* = 51; ∗∗∗*p* < 0.001. (C) Representative images of Golgi apparatus distribution in zygotes of the control and treatment groups by immunofluorescence staining. Green, Golgi; blue, DNA. Scale bars, 20 μm. (D) Relative fluorescence intensity of Golgi apparatus in zygotes. Control, *n* = 44; AlCl_3_, *n* = 44; ∗*p* < 0.05. (E) Representative images of lysosome distribution in zygotes of the control and treatment groups by immunofluorescence staining. Red, lysosome; blue, DNA. Scale bars, 20 μm. (F) Relative fluorescence intensity and abnormal distribution rate of lysosomes in zygotes. Relative fluorescence intensity: control, *n* = 50; AlCl_3_, *n* = 44; ∗∗∗*p* < 0.0005; Abnormal distribution rate: control, *n* = 50; AlCl_3_, *n* = 44; ∗∗*p* < 0.005. Data are presented as mean ± SEM. All experiments were performed in three independent biological replicates, with three technical replicates per biological replicate; mouse zygotes were used as research subjects. *n* represents the number of zygotes analyzed. Statistical analysis was performed using paired *t* tests. Asterisks indicate statistical significance: ∗*p* < 0.05, ∗∗*p* < 0.01, ∗∗∗*p* < 0.001, ∗∗∗∗*p* < 0.0001.

To assess the effect of aluminum exposure on organelle architecture in zygotes, we performed immunofluorescence staining to systematically analyze the fluorescence intensities and spatial distributions of ER, Golgi apparatus, and lysosomes. As depicted in Figures 5A and 5B, the relative fluorescence intensity in the ER was significantly increased in the AlCl_3_-treated group compared with the control group (control, 1.00 ± 0.00, *n* = 52; AlCl_3_, 1.67 ± 0.07, *n* = 51; *p* < 0.001). The Golgi apparatus is usually distributed in the region of the cytoplasm close to the nucleus. As illustrated in Figures 5C and 5D, in contrast to the control group, the AlCl_3_-treated group exhibited a diminished distribution of the Golgi apparatus both around the nucleus and throughout the cytoplasm, along with a notable reduction in the relative fluorescence intensity of the Golgi apparatus (control, 1.00 ± 0.00, *n* = 44; AlCl_3_, 0.69 ± 0.07, *n* = 44; *p* < 0.05). As depicted in Figure 5E, lysosomes in the control group were evenly dispersed throughout the cytoplasm. In contrast, lysosomes in the AlCl_3_-treated group were found to be aggregated within the cytoplasm. As shown in Figure 5F, a substantial increase in fluorescence intensity was observed in the AlCl_3_-treated group (control, 1.00 ± 0.00, *n* = 50; AlCl_3_, 2.19 ± 0.08, *n* = 44; *p* < 0.0005). Consistently, the AlCl_3_-treated group exhibited a significantly higher proportion of embryos with abnormal lysosome distribution compared to the control group (control, 31.67 ± 2.55%, *n* = 50 vs. AlCl_3_, 70.32 ± 4.89%, *n* = 44, *p* < 0.005). Collectively, these results indicate that aluminum exposure disrupts the structural organization and spatial distribution of organelles in zygotes.

## Discussion

Aluminum, a metal widely encountered in environmental exposure, represents a potential threat to reproductive health. Although its reproductive toxicity has attracted growing attention in recent years, studies specifically investigating its toxic effects on early embryonic development remain limited. In this study, an *in vitro* embryo culture model was established via IVF technology to investigate the impact of aluminum on early embryonic development. A concentration of 300 μM was selected for subsequent experiments based on preliminary dose-response analyzes.

NEBD refers to the disintegration of the nuclear envelope during mitotic prophase, whereby its lipids and proteins disperse into the cytoplasm and chromatin transitions from a relaxed to a highly condensed state, marking the cell cycle shift from interphase to mitosis.^29^ In this study, high-concentration aluminum exposure impaired NEBD, consequently leading to embryonic developmental arrest. Lamin B1, a key component of the nuclear lamina, is essential for maintaining nuclear envelope integrity and regulating cell cycle progression. Abnormal Lamin B1 function or expression in zygotes may indicate nuclear envelope structural/functional defects, thereby causing developmental arrest,^30^ which aligns with our experimental results. We observed a significant reduction in EU signal intensity, demonstrating that aluminum exposure suppresses zygotic transcriptional initiation activity. As critical transcription factors, *Dux*, *Nr5a2*, and *Sox2* play pivotal roles in regulating gene expression and chromatin architecture during early embryonic development, and their reduced expression or genetic knockout leads to developmental arrest.^31^^,^^32^ In response to oxidative stress or DNA damage, *Sox2* is upregulated to maintain cellular stemness and mitigate cellular injury.^33^ Our further analysis revealed altered expression levels of these transcription factors in aluminum-treated embryos. Collectively, these findings demonstrate that high-concentration aluminum exposure induces NEBD failure, suppresses transcriptional activity, and ultimately causes embryonic developmental arrest via disrupted nuclear envelope integrity and transcriptional dysregulation.

The cytoskeleton, comprising microtubules, microfilaments, and intermediate filaments, provides mechanical support to zygotes, maintains cellular morphology, and enables adaptive responses to developmental cues. During mitosis, microtubules assemble into the mitotic spindle to ensure accurate chromosome segregation into daughter cells.^34^ Microtubule formation initiates with nucleation, a process dependent on α-tubulin/β-tubulin heterodimers.^35^ As a fundamental subunit of microtubules, α-tubulin aberrations compromise the first zygotic mitosis, leading to erroneous chromosome segregation and subsequent developmental arrest.^36^ Prior studies show that altered acetylation levels or structural damage to microtubules disrupts their integrity and function, thereby impairing zygotic development.^37^ In our study, aluminum exposure led to a decrease in the levels of α-tubulin and its acetylation, thereby disrupting the microtubule nucleation pathway and microtubule stability, and affecting microtubule assembly. Microfilaments, primarily composed of actin, represent another cytoskeletal component.^38^ In zygotes, actin is essential for cytoskeletal architecture, cell shape maintenance, and dynamic processes like cell division.^39^ Cortical actin modulates spindle positioning through mechanical regulation, thereby indirectly ensuring pronuclear fusion and homeostasis.^40^ In contrast, cytoplasmic actin is directly involved in the transport of nuclear envelope components during pronuclear assembly.^41^ Our results further demonstrated that aluminum exposure simultaneously disrupts cortical and cytoplasmic actin. Collectively, these findings demonstrate that cytoskeletal dysfunction, mediated by the disruption of microtubules and microfilaments, leads to embryonic developmental arrest.

In early mouse embryonic development, precise cell cycle regulation is critical for ensuring orderly embryonic division and differentiation at each developmental stage.^42^ DNA damage triggers a cascade of intracellular signaling pathways, culminating in transient or permanent cell-cycle arrest. As a canonical DNA damage marker, nuclear accumulation of γ-H2A.X typically indicates DNA lesions within the cell.^43^ After the occurrence of DNA double-strand breaks (DSBs), the *Atm/Atr* kinases, core upstream molecules in the DDR, must first be activated.^44^ These kinases then phosphorylate the downstream substrate H2A.X to form γ-H2A.X, which in turn recruits repair factors such as *Brca1* to assemble a DNA damage repair complex.^45^ Our findings demonstrated that the mRNA expression levels of *Atm*, *Atr*, and *Brca1* were significantly decreased at 12 h postfertilization (hpf), which directly led to insufficient activation of these upstream kinases, failure to effectively initiate the phosphorylation of H2A.X, a reduction in γ-H2A.X production, and a consequent decrease in its fluorescence intensity. RAD51 is a core recombinase central to homologous recombination (HR) repair, which mediates the pairing and strand exchange between homologous DNA molecules during error-free DSB repair.^46^ The reduced expression of this protein impairs the efficient recruitment of downstream repair factors and the subsequent formation of stable damage-site complexes, thereby rendering γ-H2A.X vulnerable to dephosphorylation and degradation by phosphatases.^47^ This finding explains the reduced γ-H2A.X fluorescence intensity observed in aluminum-exposed mouse zygotes at 12 hpf, despite the presence of DNA oxidative damage. As a cell cycle checkpoint kinase that plays a pivotal role in orchestrating cell cycle progression in response to DNA damage, a reduction in CHK1 fluorescence intensity abrogates cell cycle checkpoint function, thereby forcing cells with unrepaired DNA lesions to initiate apoptotic programs prematurely and shortening the time window required for γ-H2A.X signal maintenance.^48^ Collectively, the dysregulation of both RAD51 and CHK1 further attenuates the γ-H2A.X fluorescent signal. Furthermore, 8-OHdG, a core marker of oxidative DNA damage, accumulates to exacerbate DNA lesions^49^; yet, the obstruction of repair pathways and the induction of apoptosis impede the efficient amplification of damage signals, leading ultimately to a reduction in γ-H2A.X fluorescence intensity.^50^ This is consistent with our results. Unrepaired DNA damage induces G2/M checkpoint activation to inhibit mitotic entry of damaged cells, with CDC25C-mediated phosphorylation essential for the activity of CDK1/Cyclin B1, the core G2/M transition switch.^51^ In our study, the synchronous downregulation of CDC25C, CDK1, and Cyclin B1 directly led to the loss of complex function, impaired the progression of cells from G2 to M phase, and ultimately induced G2/M phase arrest. Furthermore, *Myt1* suppresses CDK1 function via phosphorylation, thereby inducing irreversible cell-cycle arrest.^52^^,^^53^ Altogether, these findings demonstrate that aluminum exposure disrupts the DDR-CHK1-CDK1 axis, leading to G2/M arrest via integrated mechanisms of cell cycle dysregulation and apoptotic activation.

Mitochondria, serving as the cellular powerhouse, are essential for energy production and intricately linked to cell survival-death decisions.^54^ Compromised mitochondrial function disrupts the balance between ROS production and scavenging, leading to oxidative stress-mediated damage of cellular biomacromolecules and triggering apoptosis or necrosis.^55^ Our findings demonstrate that aluminum exposure induces aberrant mitochondrial distribution and a reduction in mitochondrial membrane potential in mouse zygotes, indicative of mitochondrial dysfunction. Furthermore, we observed that the ROS-positive rate was significantly elevated at 5.5 hpf, followed by a marked decline at the subsequent time points of 6, 9, and 12 hpf. Short-term aluminum exposure mildly inhibits the activity of electron transport chain complexes, thereby increasing the release of mitochondria-derived ROS,^56^ which is consistent with our findings. Following an acute ROS burst, cells initiate compensatory antioxidant defenses to attenuate the persistent generation of mitochondria-derived ROS.^57^ However, this compensatory response appears to be transient and insufficient in zygotes under prolonged aluminum stress. In our study, at 12 hpf, abnormal mitochondrial distribution was observed, accompanied by a decrease in TMRE fluorescence intensity and a downregulated mRNA expression level of *Cox1*. As the primary site of cellular ROS production, mitochondria rely on Complex IV for efficient electron transport, and given that cox1 encodes a core subunit of this complex, its downregulation is likely a key contributor to respiratory chain dysfunction and subsequent membrane potential collapse, which directly blocks electron leakage, thereby leading to a marked reduction in mitochondria-derived ROS generation.^58^ Meanwhile, the decreased mRNA expression levels of antioxidant system-related genes such as *Sod1* and *Sod2* indicate that long-term aluminum exposure has impaired the activity of antioxidant enzymes due to continuous consumption. Additionally, the occurrence of apoptosis in zygotes at 12 hpf affects the targeting and activation efficiency of ROS probes. Simultaneously, the impaired membrane integrity of some apoptotic cells may cause probe leakage, which further exacerbates the false decrease in ROS fluorescence signal.^59^ 8-OHdG is a specific biomarker of oxidative DNA damage,^60^ whereas 4-HNE is a hallmark product of lipid peroxidation^61^; although ROS itself has an extremely short half-life, the irreversible oxidative lesions it induces (including lipid peroxidation and DNA oxidation) can persist intracellularly and thus be readily detected,^62^ a phenomenon that explains the elevated levels of 4-HNE and 8-OHdG we detected at 12 hpf, even when ROS levels were diminished. Notably, decreased ROS levels, as a consequence of respiratory chain dysfunction, further exacerbate the decline in mitochondrial membrane potential and the opening of mitochondrial permeability transition pores (mPTPs), thereby promoting the release of cytochrome *c* and initiating the mitochondrial apoptotic pathway.^63^ Meanwhile, reduced ROS downregulates the expression of the anti-apoptotic protein *Bcl2*, thus disrupting the intracellular anti-apoptotic barrier.^64^ Although the mRNA expression level of the pro-apoptotic protein *Bax* is decreased, the proportion of free and active *Bax* protein still increases due to the weakened binding between *Bcl2* and *Bax*.^65^ Additionally, the upregulated expression of *Jun* protein further activates downstream pro-apoptotic genes, amplifying the apoptotic signal.^66^ Taken together, aluminum exposure impairs the normal physiological function of mitochondria in zygotes, leading to the massive accumulation of oxidative damage products and ultimately mediating the occurrence of cellular apoptosis.

DNA damage and apoptosis disrupt intracellular proteostasis and redox balance, leading to protein misfolding, ER dysfunction, and subsequent ER stress response.^67^ Concurrently, this imbalance disrupts cytoskeletal dynamics, altering the structure and function of microtubules and microfilaments, which in turn impairs Golgi apparatus positioning.^68^ Additionally, DNA damage-induced cell-cycle arrest (e.g., G2/M phase arrest) influences Golgi distribution, as its function is tightly coupled to the cell cycle.^69^ Apoptotic signals compromise lysosomal membrane integrity, releasing hydrolases into the cytoplasm,^70^ while ER stress inhibits lysosomal enzyme synthesis and folding.^71^ Concomitantly, Golgi dysfunction disrupts lysosomal enzyme modification and transport.^72^ We found that aluminum exposure disrupts the function of the ER, Golgi apparatus, and lysosomes in zygotes. Studies have shown that aluminum exposure impairs the quality of mouse oocytes by disrupting the distribution and function of the ER, Golgi apparatus, and lysosomes.^18^ In summary, these findings confirm our hypothesis that aluminum exposure leads to embryonic developmental arrest by causing dysfunction of the ER, Golgi apparatus, and lysosomes within the embryo.

In summary, aluminum exposure disrupts the cytoskeletal integrity and cell cycle progression of zygotes, which may be attributed to mitochondrial dysfunction-based oxidative damage and apoptosis for the failure of NEBD.

### Limitations of the study

This study adopted an *in vitro* embryo aluminum exposure model, which has certain limitations compared with the complex physiological microenvironment *in vivo*. The analysis of this study mainly focused on core indicators such as mitochondrial function, oxidative stress, DNA damage, histone modification, and organelle distribution. Currently, the exploration of the downstream regulatory pathways and molecular network mechanisms by which aluminum exposure affects embryo development still has room for further in-depth research. In the future, an *in vivo* aluminum exposure animal model can be established, and on this basis, combined with multi-omics technologies, differentially expressed genes, proteins, and epigenetic modification sites in embryo tissues after aluminum exposure can be systematically screened to deeply analyze the molecular networks and signaling pathways of embryo development regulated by aluminum exposure.

## Resource availability

### Lead contact

Requests for further information and resources should be directed to and will be fulfilled by the lead contact, Lin-Lin Hu (hutwolin@126.com).

### Materials availability

This study did not generate new unique reagents.

### Data and code availability

• Data reported in this paper will be shared by the lead contact upon request.
• This paper does not report original code.
• Any additional information required to reanalyze the data reported in this paper is available from the lead contact upon request.

## Acknowledgments

We would like to acknowledge the contributions from all individuals who provided technical assistance, support, and valuable suggestions during the research process but are not listed as authors. This work was supported by the Natural Science Foundation of Guangxi in China (2025GXNSFAA069655, 2025GXNSFAA069209); the 10.13039/501100012226Fundamental Research Funds for the Central Universities of China (KYT2024002, KJJQ2025001, RENCAI2024011); the 10.13039/501100001809National Natural Science Foundation of China (82360292); and the Scientific Research and Technological Development Foundation of Baise (20224113). We sincerely thank all the funding bodies for their financial support.

## Author contributions

S.-C.S. and L.-L.H. designed the study; X.-T.Y. performed the experiments; X.-H.K., Z.-J.W., Z.-H.F., R.-J.M., X.W., and M.-M.T. contributed the materials; X.-T.Y. wrote the manuscript; X.-T.Y. and S.-C.S. analyzed the data. All the authors approved the final manuscript.

## Declaration of interests

The authors declare no competing interests.

## STAR★Methods

### Key resources table

**Table:** 

REAGENT or RESOURCE	SOURCE	IDENTIFIER
**Antibodies**		

anti-α-tubulin-FITC antibody	Sigma-Aldrich	Cat# F2168; RRID: AB_476967
Anti-acetylated-tubulin antibody	Cell Signaling Technology	Cat# 5335; RRID: AB_10544694
β-Actin	Cell Signaling Technology	Cat# 3700; RRID: AB_10985704
TRITC Phalloidin	SAITONG	Cat# T10446-300T; RRID: N/A
Anti-gamma H2A.X antibody	Abcam	Cat# ab81299; RRID: AB_1640564
Anti-Chk1 antibody	Abcam	Cat# ab40866; RRID: AB_726820
Anti-RAD51 antibody	Proteintech	Cat# 14961-1-AP; RRID: AB_2177083
Anti-4-Hydroxynonenal antibody	Proteintech	Cat# 68538-1; RRID: AB_3085246
Anti-CDC25C antibody	Proteintech	Cat# 66912-1; RRID: AB_2882239
Anti-Lamin B1 antibody	Proteintech	Cat# 12987-1-AP; RRID: AB_2136290
Anti-phospho-CDK1 (Thr161) antibody	HuaBio	Cat# HA721987; RRID: N/A
Tubulin	Abways	Cat# AB0049; RRID: N/A
8-OHdG antibody	MedChemExpress	Cat# HY-P81140; RRID: AB_3103069
TRITC-conjugated goat anti-rabbit IgG H&L	Zsbio	Cat# ZF-0316; RRID: AB_2571576
Alexa Fluor 488-conjugated goat anti-rabbit IgG H&L	Zsbio	Cat# ZF-0511; RRID: AB_2534102
Alexa Fluor 594-conjugated goat anti-mouse IgG H&L	Zsbio	Cat# ZF-0513; RRID: AB_2534068
Alexa Fluor 488-conjugated goat anti-mouse IgG H&L	Zsbio	Cat# ZF-0512; RRID: AB_2534084

**Chemicals, peptides, and recombinant proteins**		

AlCl3·6H_2_O	Sigma-Aldrich	Cat# 7784-13-6
Pregnant mare serum gonadotropin (PMSG)	Ningbo Sansheng Pharmaceutical	Cat# 110254564
Human chorionic gonadotropin (hCG)	Ningbo Sansheng Pharmaceutical	Cat# 110251282
Hoechst 33342	Sigma-Aldrich	Cat# B2261
HTF medium	Nanjing Luanchuang Life Technology Company	Cat# M04-F
TYH medium	Nanjing Luanchuang Life Technology Company	Cat# M05-Y
KSOM medium	Nanjing Luanchuang Life Technology Company	Cat# M03-AA
Mito-tracker red CMXRos	Thermo Fisher	Cat# M7512
LysoTracker red	Beyotime	Cat# C1046
Golgi-tracker green	Beyotime	Cat# C1043
DCFH-DA	Beyotime	Cat# S0033
HiScript III RT SuperMix for qPCR	Invitrogen	Cat# R323-01
4% Paraformaldehyde	solarbio	Cat# P1110
SYBR qPCR Master Mix	Invitrogen	Cat# Q711-02

**Critical commercial assays**		

Mitochondrial Membrane Potential Assay Kit with TMRE	Beyotime	Cat# C2001
Annexin V-FITC Apoptosis Detection Kit	Beyotime	Cat# C1062M
Cell-Light™ EU Apollo488 RNA Imaging Kit	RiboBio	Cat# C10316-3
Dynabeads™ mRNA DIRECT™	Invitrogen	Cat# 61011

**Deposited data**		

All data supporting the findings of this study	This paper	No accession codes; data available within article and supplemental information

**Oligonucleotides**		

Primers for qRT-PCR, see Table S1	This paper	N/A

**Software and algorithms**		

GraphPad Prism software	N/A	GraphPad Prism 9.5
ImageJ	N/A	https://imagej.net/
ZEN	N/A	ZEN 2012

**Other**		

Original western blot images, see Figure S1	This paper	N/A

### Experimental model and study participant details

#### Animals

All animal experiments in this research were approved by the Animal Care and Utilization Committee of Nanjing Agricultural University (Ethical Approval No.: NJAU. No20241223268) and were carried out strictly in accordance with the requirements of the guidelines of the Animal Research Committee. All experiments in this study were conducted using SPF-grade ICR mice. Female SPF-grade ICR mice (6–8 weeks old) were purchased from Nanjing Medical University, and male SPF-grade ICR mice (10–11 weeks old) were purchased from Jiangsu Qinglongshan Biotechnology Co., Ltd. Female mice were super-ovulated for the collection of cumulus-oocyte complexes (COCs), and sperm were collected from male mice for *in vitro* fertilization (IVF). Following IVF and *in vitro* culture, zygotes and preimplantation embryos were obtained, and all study subjects in this study were zygotes obtained by IVF.

All mice were housed in the Animal Experiment Center of Nanjing Agricultural University under SPF conditions with controlled temperature (24 ± 1°C), humidity (50 ± 10%), and a 12-h light/dark cycle. Animals were provided *ad libitum* access to standard chow and water, and cage bedding was changed twice weekly. Mice were acclimated to the facility for at least one week before experimental procedures. Embryo manipulations and *in vitro* culture were performed in appropriate medium under mineral oil at 37°C with 5% CO_2_.

Since this study focused on early preimplantation embryos without distinguishing sex, sex and gender were not considered as biological variables, and no sex-specific effects were analyzed in the present work.

### Method details

#### *In vitro* fertilization and embryo culture

Female mice were primed with 10 IU of PMSG to promote follicular development. 46 to 48 h later, 10 IU of hCG was administered to induce ovulation. 13 to 15 h after hCG injection, sperm was collected from the cauda epididymis of male mice and capacitated in TYH medium for 1 h. One hour later, COCs were harvested from the ampullary region of the female mouse fallopian tubes and transferred to HTF medium. Sperm was then added to the HTF medium, and the mixture was cultured at 37 °C in a 5% CO_2_ incubator for 4–6 h. Zygotes were washed and transferred to KSOM medium for subsequent culture until reaching the 2-cell stage. All experiments in this study were conducted using ICR mice.

#### AlCl_3_ treatment

A 50 mM conversion solution was prepared by dissolving AlCl_3_ in water. This solution was then diluted into KSOM medium to obtain working solutions with final concentrations of 100 μM, 200 μM, and 300 μM.^73^^,^^74^ We evaluated the developmental rates of embryos exposed to these concentrations and selected 300 μM for subsequent experiments based on the observed results.

#### Immunofluorescence staining and confocal microscopy

At 12 h postfertilization, zygotes were fixed in 4% paraformaldehyde at room temperature for 30 min. Embryos were then permeabilized with 0.5% Triton X-100 for 20 min at room temperature, followed by blocking in 1% BSA-supplemented PBS for 1 h at room temperature or overnight at 4°C. Subsequently, embryos were incubated with primary antibodies against Lamin B1 (1:300), acetylated tubulin (1:200), anti-α-tubulin-FITC (1:200), actin (1:200), γ-H2A.X (1:250), and CHK1 (1:200), 8-OHdG (1:200), and 4-HNE (1:800) overnight at 4°C. After three washes in PBS containing 0.1% Tween 20 and 0.01% Triton X-100 (5 min each), embryos were incubated with corresponding secondary antibodies for 1 h at room temperature. Following three additional washes, chromosomes were stained with Hoechst 33342, and embryos were observed using a Zeiss LSM 700 META laser-scanning confocal microscope.

#### EU labeling and staining

At 5 h postfertilization, zygotes were transferred to KSOM medium supplemented with 5-ethynyl uridine (EU, 1:1000) and incubated at 37°C in 5% CO_2_ for 7 h. Following EU incorporation, embryos were fixed in 4% paraformaldehyde for 30 min at room temperature, permeabilized with 0.5% Triton X-100 for 20 min, and blocked with 1% BSA-supplemented PBS for 1 h at room temperature or overnight at 4°C. Subsequently, embryos were incubated in 1X Apollo staining reaction solution for 45–60 min at room temperature to detect freshly synthesized RNA. After three washes in PBS containing 0.1% Tween 20 and 0.01% Triton X-100 (5 min per wash), nuclei were stained with Hoechst 33342 for 10 min at room temperature. Embryos were then mounted for imaging using laser confocal microscopy.

#### Mitochondria, ROS, lysosome and ER staining in living embryos

Prepare staining solutions by diluting Mito-Tracker (1:600), ROS indicator (1:800), Lyso-Tracker (1:10,000), ER-Tracker (1:600), and Hoechst 33342 (1:200) in pre-warmed KSOM medium. Equilibrate the solutions in a 37°C, 5% CO_2_ incubator for 30 min. At 12 h postfertilization, transfer zygotes into the staining solution and incubate for 30 min. Following incubation, wash zygotes three times with fresh KSOM medium. Finally, mount zygotes on a live-cell imaging dish and visualize them using a laser-scanning confocal microscope with appropriate excitation/emission filters.

#### Annexin-V staining

Zygotes were incubated in Annexin V-FITC apoptosis detection reagent (1:10 dilution in binding buffer) for 18 min at 37°C in 5% CO_2_. Following incubation, zygotes were fixed in 4% paraformaldehyde for 30 min at room temperature, permeabilized with 0.5% Triton X-100 for 20 min, and blocked in 1% BSA-supplemented PBS for 1 h at room temperature or overnight at 4°C. Finally, the zygotes were examined using a laser confocal microscope.

#### Western blot analysis

A minimum of 80 zygotes were collected per group and lysed in Laemmli sample buffer. The samples were then boiled at 100°C for 10 min and stored at −20°C. Subsequently, the samples were subjected to SDS-polyacrylamide gel electrophoresis (PAGE) at 160 V for approximately 70 min. Proteins were transferred onto polyvinylidene difluoride (PVDF) membranes, which were then blocked with a rapid blocking solution for 15 min at room temperature. The membrane was incubated with primary antibodies against CDC25C (1:1000), *p*-CDK1(T161) (1:1000), Cyclin B1 (1:1000), α-tubulin (1:1000), and β-actin (1:1000) at 4°C for one day. The membrane was then washed three times with TBST for 10 min each time and incubated with the secondary antibody at room temperature for 1 h. After three additional washes with TBST, the PVDF membrane was treated with an enhanced chemiluminescence reagent, and the protein bands were visualized using the Tanon-3900 system. Finally, the relative signal intensities were quantified using ImageJ software.

#### Quantity real-time PCR (qRT-PCR)

At least 20 zygotes were collected 12 h postfertilization. RNA was extracted using the magnetic bead method, reverse transcribed into cDNA, and stored at −20°C. A 20 μL qPCR reaction mixture was prepared. The mRNA levels were quantified by Real-Time quantitative polymerase chain reaction (qPCR) using the One plus real-time PCR system, with each experiment performed at least three times. The relative mRNA levels were subsequently determined using the 2^−ΔΔCt^ method.

### Quantification and statistical analysis

Each experiment was performed with at least three biological replicates, with three technical replicates per biological replicate; mouse zygotes were used as research subjects, and each sample group contained no fewer than 30 zygotes. The control and experimental groups were subjected to identical experimental conditions. Fluorescence intensity was analyzed using ZEN lite 2012 and ImageJ software, while data analysis was performed with GraphPad Prism 9.5. For Lamin B1, the outermost edge of the pronucleus was defined as the region of interest (ROI). For EU and γ-H2A.X, the entire pronuclear region was designated as the ROI. For cortical actin, the cortical layer at the outermost edge of the zygote was set as the ROI. For α-tubulin, acetylated α-tubulin (Ac-tubulin), cytoplasmic actin, CHK1, mitochondria, TMRE, ROS, 8-OHdG, 4-HNE, endoplasmic reticulum (ER), Golgi apparatus, and lysosomes, the whole zygote was defined as the ROI. The average fluorescence intensity per unit area within each ROI was calculated using ImageJ software. All imaging parameters (excitation power, exposure time, gain) were kept consistent across all experimental and control groups to avoid artificial differences in fluorescence intensity. Furthermore, all image analysis was performed in a blinded manner, where the analyst was unaware of the treatment group assignments of the samples. For normalization, relative fluorescence intensity was defined as the fold change relative to the control group, calculated by dividing the mean intensity of the treatment group by the mean intensity of the control group. Statistical analysis was performed using paired t-tests. Data are presented as mean ± SEM, with n representing the number of zygotes analyzed. Asterisks indicate statistical significance: ∗*p* < 0.05, ∗∗*p* < 0.01, ∗∗∗*p* < 0.001, ∗∗∗∗*p* < 0.0001.
