# Supplementary material for: Aluminum exposure impairs nuclear envelope breakdown for mouse zygote formation
Source: iScience. 2026 Apr 21;29(5):115807. doi: 10.1016/j.isci.2026.115807 (PMC13157191; doi:10.1016/j.isci.2026.115807)
Supplement: Document S1. Figure S1 and Table S1 [file mmc1.pdf]

iScience, Volume *29*

Supplemental information

Aluminum exposure impairs nuclear envelope breakdown for mouse zygote formation

Xiao-Ting Yu, Xing-He Ke, Zi-Jian Wu, Zhen-Hui Fu, Rui-Jie Ma, Xuan Wu, Meng-Meng Tang, Shao-Chen Sun, and Lin-Lin Hu

Supplementary figures

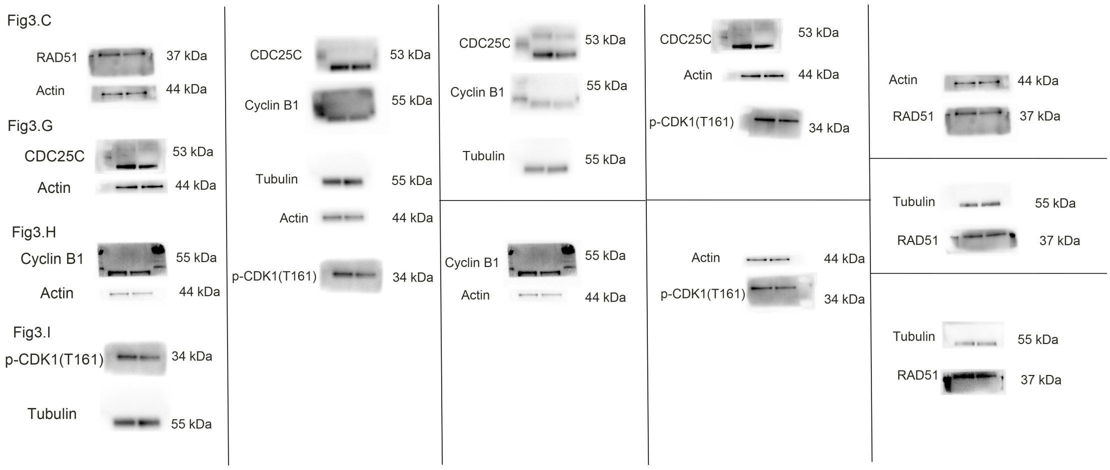

Figure S1. Uncropped original Western blot images, related to Figure 3

Uncropped original Western blot images for the detection of target and reference proteins (RAD51, CDC25C, Cyclin B1, p-CDK1 (T161), Actin, Tubulin) are shown. Molecular weight markers (kDa) for all protein bands are indicated on the blot as referenced. All Western blot experiments were independently repeated three times to ensure reproducibility, and the cropped blot images used in the main text are derived from these full-length original blots. Antibody information for all detected proteins is detailed in the Key Resources Table of the main manuscript.

Table S1: Primer sequences used in qRT-PCR analysis, related to STAR Methods.

Gene	Forward Primer	Reverse Primer
Gapdh	AGGTCGGTGTGAACGGATTTG	TGTAGACCATGTAGTTGAGGTCA
Dux	CCCAGCGACTCAAACTCCTTC	GGACTTCGTCCAGCAGTTGAT
Nr5a2	TGAGGAACAACTCCGGGAAAA	CAGACACTTTATCGCCACACA
Sox2	GCGGAGTGGAAACTTTTGTCC	CGGGAAGCGTGTACTTATCCTT
Pten	TGGATTCGACTTAGACTTGACCT	GCGGTGTCATAATGTCTCTCAG
Cdkn1b	TCAAACGTGAGAGTGTCTAACG	CCGGGCCGAAGAGATTTCTG
Myt1	TGCAGACCTCAGTTGTCCTAC	TCCTCTTGGATACCAGGTGCT
Apc2	CACACAGTTTGACCATCGTGA	GTGGACGAGGTTGCGTAGC
Brca1	CGAATCTGAGTCCCCTAAAGAGC	AAGCAACTTGACCTTGGGGTA
Atm	GATCTGCTCATTTGCTGCCG	GTGTGGTGGCTGATACATTTGAT
Atr	GAATGGGTGAACAATACTGCTGG	TTTGGTAGCATACACTGGCGA
Gpx1	AGTCCACCGTGTATGCCTTCT	GAGACGCGACATTCTCAATGA
Sod1	AACCAGTTGTGTTGTCAGGAC	CCACCATGTTTCTTAGAGTGAGG
Sod2	CAGACCTGCCTTACGACTATGG	CTCGGTGGCGTTGAGATTGTT
Cat	GCAGATACCTGTGAACTGTC	GTAGAATGTCCGCACCTGAG
Cox1	GCTCTTTCATGCCCTAATCTTCTAC	GGAGGTGTTGAGGGTTTGGTTATG
Bcl2	GTCGCTACCGTCGTGACTTC	CAGACATGCACCTACCCAGC
Bax	TGAAGACAGGGGCCTTTTTG	AATTCGCCGGAGACACTCG
Jun	CCTTCTACGACGATGCCCTC	GGTTCAAGGTCATGCTCTGTTT
